# Mechanistic origin of high-cycle fatigue enhancement by grain refinement in AZ81 magnesium alloy for sports equipment

**DOI:** 10.1371/journal.pone.0350435

**Published:** 2026-06-04

**Authors:** Dong Li, Liuyong He

**Affiliations:** 1 Huanghuai University, Zhumadian, China; 2 School of Mechanical and Power Engineering, Henan Polytechnic University, Jiaozuo, Henan, China; Lovely Professional University, INDIA

## Abstract

Lightweight, high-strength alloys are increasingly demanded in sports equipment. Magnesium (Mg) alloys are attractive due to their low density and high specific strength. This study systematically investigates the grain size–dependent high-cycle fatigue (HCF) behavior of AZ81 Mg alloy with comparable basal textures. The fine-grained (FG, ~ 8 μm) sample exhibits significantly improved mechanical performance compared with the coarse-grained (CG, ~ 62 μm) counterpart. The yield strength increases from 134.2 MPa to 164.5 MPa (~22.6%), and the ultimate tensile strength rises from 231.7 MPa to 283.7 MPa (~22%), while maintaining comparable ductility. More importantly, the fatigue strength at 10⁶ cycles increases from 80 MPa to 110 MPa, representing a 37.5% enhancement. Microstructural analyses reveal that grain refinement suppresses extension twinning and persistent slip band formation, while promoting the activation of <c + a> and non-basal <a> dislocations. The FG microstructure also contains finer and more uniformly distributed Mg_17_Al_12_ precipitates, facilitating Orowan strengthening. These combined effects reduce strain localization and delay fatigue crack initiation. The findings clarify the mechanistic origin of grain refinement–induced fatigue enhancement and provide guidance for the design of Mg alloys in weight-critical sports applications.

## 1. Introduction

In the sports equipment industry, there is a constant drive to use lightweight high-strength alloys in structural components such as bicycle frames, racket heads, and automotive sports parts to improve agility and energy efficiency [1–3]. Magnesium (Mg) alloys are of particular interest because of their low density (about 1.8 g/cm³, ~ 2/3 that of aluminum (Al)) combined with relatively high specific strength [4,5]. AZ-series Mg alloys (Mg-Al-Zn system) are widely used in engineering due to their good castability and balanced mechanical properties. However, sports equipment components (e.g., bike frames, golf club heads) experience repetitive loading [6], understanding and improving the fatigue behavior of Mg alloys is therefore essential.

Fatigue performance of Mg alloys is highly sensitive to their microstructure (e.g., grain size, texture, second phases, et al.) [6–9]. Prior studies have shown that metallurgical factors like grain refinement and precipitate distribution can dramatically influence fatigue life, especially in the high-cycle (low-stress) regime [8,10]. Grain size refinement is a well-known strengthening strategy, reducing grain size increases the yield strength (YS) via the Hall–Petch relationship [11], which often also improves high-cycle fatigue strength due to the linkage between static and dynamic strength [12,13]. For example, refined AZ91 alloy grain structure (through thermomechanical processing or alloying additions) has been reported to enhance its fatigue endurance limit by reducing internal stress concentrations and blocking slip propagation [14,15]. Fine grains introduce more grain boundary area that can impede dislocation motion (increasing strength) and can also promote more uniform plastic deformation, delaying crack initiation under cyclic loads [16–18]. While grain refinement is generally seen as beneficial for Mg alloy fatigue, the slip mode activation and dynamic microstructural evolution remain unclear. In this work, we systematically correlate fatigue crack-initiation modes with dislocation configurations in AZ81 alloy, revealing how fine grains alter the damage process.

Previous studies on pure Mg [19], AZ31 [11,20], AZ91 [15,21], and rare earth Mg [22,23] alloys have demonstrated that grain refinement can enhance fatigue strength by increasing YS and suppressing strain localization. However, contradictory observations also exist, particularly when the grain size approaches the ultrafine regime, where limited strain hardening capacity and cyclic instability may counteract the strengthening benefits [24,25]. Malekjani et al. [26] noted that while grain refinement markedly increased the HCF life of pure Al, it did not improve (and could degrade) low-cycle fatigue (LCF) life due to reduced ductility and microstructural instability under large cyclic strains. These results highlight the need for a nuanced understanding of the grain size–fatigue relationship, moderate grain refinement can be beneficial, however other factors (e.g., dislocation storage ability, twinning, and stability of microstructure under cycling) come into play. Although previous studies have reported the influence of grain refinement on fatigue strength in Mg alloys [15,27], most investigations have primarily focused on macroscopic fatigue performance without systematically correlating grain size to specific cyclic deformation mechanisms under controlled texture conditions. In particular, for AZ81 Mg alloy, the interplay among grain size, non-basal slip activation (especially ⟨c + a⟩ dislocations), strain localization behavior, and fatigue crack initiation remains insufficiently understood.

In addition, due to the hexagonal close-packed (HCP) crystal structure of magnesium, plastic deformation is strongly anisotropic and highly dependent on the activation of limited slip systems [28]. Under cyclic loading, basal <a> slip is typically dominant because of its low critical resolved shear stress (CRSS), while extension twinning when the ***c***-axis of the grain be compressed [29]. Persistent slip band (PSB) formation and twin boundary interactions are frequently reported as primary fatigue crack initiation sites in extruded Mg alloys. However, the activation of non-basal slip systems, particularly ⟨c + a⟩ dislocations, has been suggested to improve strain compatibility and reduce deformation heterogeneity during cyclic loading [29]. Despite this understanding, the conditions under which grain refinement promotes such mechanisms remain insufficiently clarified.

The present study distinguishes itself by isolating the grain size effect under comparable basal texture conditions and establishing a microstructure–mechanism–fatigue strength relationship. By combining EBSD-based strain mapping, crack statistics, and TEM characterization, this work elucidates how grain refinement promotes ⟨c + a⟩ activation, enhances strain compatibility, and suppresses crack initiation through both deformation coordination and dynamic precipitation mechanisms. This integrated mechanistic understanding provides new insight into grain size–dependent fatigue enhancement in AZ81 alloy.

## 2. Experimental methods

### 2.1. Material preparation

The AZ81 Mg alloy (nominal composition Mg–8wt%Al–1wt%Zn–0.2wt%Mn) was acquired as cast billets. To obtain two distinct grain sizes with comparable texture, a two-step thermo-mechanical processing route was employed. First, the as-cast billet was hot-extruded at 350 °C into plates (extrusion ratio ~12:1) to introduce a uniform basal texture, following the processing conditions reported in previous studies on AZ81 alloy [30]. This yielded a fine-grained (FG) microstructure through dynamic recrystallization. A portion of the extruded plates was then subjected to a grain growth heat treatment at 420 °C for 4 h, and rapidly cooled by water quenching to produce a coarse-grained (CG) microstructure, consistent with protocols used in related Mg-Al-Zn alloy investigations [31,32]. The annealing parameters were selected to promote static grain growth while minimizing texture evolution, as reported in previous studies on extruded Mg–Al–Zn alloys. Under such conditions, grain coarsening occurs primarily through boundary migration without significant crystallographic reorientation, allowing the basal texture introduced during extrusion to be largely retained. This approach enables a controlled comparison of grain size effects on fatigue behavior without the interference of texture variation. By this method, both FG and CG samples share a similar crystallographic texture (strong basal planes parallel to the extrusion axis) but have different grain sizes.

### 2.2. Mechanical testing

For mechanical testing, standard dog-bone specimens were machined from the extruded plates along the extrusion direction (ED) for both tensile and fatigue tests. The gauge dimensions were 8 mm (length) × 4 mm (width) × 5 mm (thickness), with the loading direction parallel to the ED. All sample surfaces were polished to remove machining marks and to reduce any surface effects on fatigue. Quasi-static tensile tests were conducted at room temperature in accordance with ASTM E8/E8M standards using an Instron universal testing machine at a strain rate of 1 × 10 ^−^ ³ s ^−^ ¹. Three tests for each grain size condition provided yield strength (YS) (0.2% offset), ultimate tensile strength (UTS), and elongation (EL) to failure values. High-cycle fatigue tests were performed under force-controlled axial loading in accordance with ASTM E466, using a servo-hydraulic fatigue testing system. A sinusoidal waveform was applied at a frequency of 30 Hz. To simulate alternating stress conditions similar to sports equipment in use, a stress ratio of R = −1 (fully reversed cycling) was chosen for tests. At each stress amplitude level, at least three specimens were tested to ensure reproducibility. The stress amplitudes were selected based on the tensile properties of the material and adjusted in a stepwise manner to efficiently determine the fatigue limit. A specimen was considered failed when complete fracture occurred. Specimens that survived up to 10⁶ cycles without failure were defined as run-outs. The fatigue limit was estimated as the maximum stress amplitude at which all tested specimens survived 10⁶ cycles without failure. All tests were conducted under ambient laboratory conditions (~25 °C, relative humidity ~50%). To investigate the microstructural evolution during fatigue, interrupted tests were conducted under comparable fractions of fatigue life, ensuring that the examined microstructures correspond to similar damage states. After interruption, the specimens were immediately unloaded to zero stress to prevent additional deformation. EBSD and TEM samples were extracted from regions near the gauge section.

### 2.3. Microstructural characterization

This work employed a scanning electron microscope (SEM) equipped with electron backscattered diffraction (EBSD) system for microstructural characterization. Samples were sequentially ground using 400#, 1000#, 2000#, and 3000# abrasive papers followed by mechanical polishing to achieve a scratch-free mirror surface. For microstructural observation, specimens were etched with 10% diluted nitric acid for 1 s, immediately rinsed with water to prevent over-etching, then cleaned with alcohol and dried [33]. Prior to EBSD analysis, samples were similarly ground and mechanically polished, then electro-phished in AC Ⅱ solution at −30 °C to −25 °C under 20 V for 150 s, followed by immediate alcohol rinsing and storage in alcohol until drying before observation. Transmission electron microscopy (TEM) was performed using a JEOL 2100 microscope operating at 200 kV to characterize dislocations and precipitate phases. TEM specimen preparation involved sectioning 0.5 mm-thick slices from regions of interest, grinding to 50 μm thickness using the same abrasive paper sequence, punching 3 mm discs, and final thinning with a Gatan 695 precision ion polishing system. Convergent beam electron diffraction (CBED) was used to profile the thickness of TEM foils [27].

## 3. Results and discussion

### 3.1. Initial microstructure and texture

**Fig 1:**
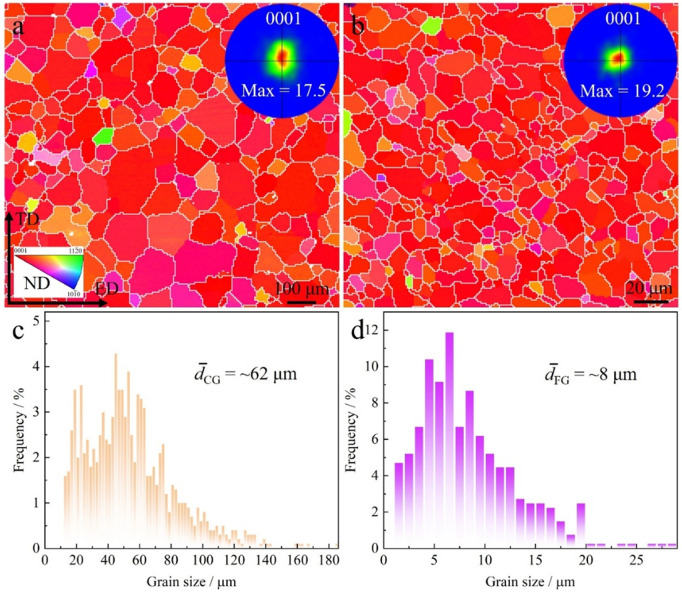
Initial microstructures of the AZ81 samples: EBSD IPF and PF images of the (a) CG and (b) FG samples, (d) the distribution of grain size of the (c) CG and (d) FG samples.

Fig 1 presents the EBSD maps and grain size distributions of CG and FG AZ81 Mg alloys. As shown in Fig 1a and b, both CG and FG samples exhibit a strong texture, with maximum pole densities of 17.5 and 19.2, respectively, indicating the presence of a typical extrusion-induced basal texture. Although the FG sample shows a slightly higher orientation density, the difference is minor, suggesting that the grain refinement process during heat treatment did not significantly alter the texture characteristics. Therefore, when comparing the mechanical behaviors of the two samples, the influence of texture on deformation behavior is limited (S1 Fig), and grain size can be considered the dominant variable. The observed strong basal texture in both CG and FG samples is consistent with previous EBSD studies on extruded Mg–Al–Zn alloys, where basal planes are preferentially aligned parallel to the extrusion direction [34]. Moreover, the negligible variation in texture intensity after grain growth heat treatment agrees well with earlier reports, which indicate that static grain growth primarily affects grain size without significantly modifying the crystallographic texture. The grain size distributions are shown in Fig 1c and d. The CG sample exhibits a broad grain size distribution mainly concentrated in the 40–60 μm range, with a substantial number of grains exceeding 100 μm, resulting in an average grain size of approximately 62 μm. In contrast, the FG sample shows a much narrower distribution, primarily in the 5–10 μm range, with an average grain size of 8 μm. These results clearly demonstrate that a significant grain refinement was successfully achieved through the applied heat treatment process without degrading the crystallographic texture. This microstructural transition provides an ideal model system for investigating the effect of grain size on fatigue mechanisms in subsequent studies.

### 3.2. Mechanical performance

**Fig 2:**
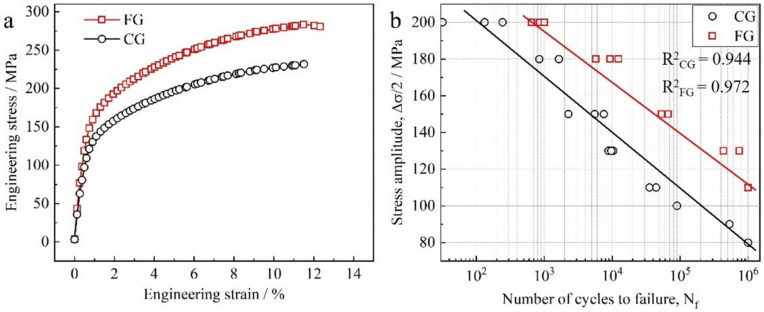
Tensile and high-cycle fatigue properties of the CG and FG samples: (a) Engineering stress-strain curve, (b) S-N curve.

Fig 2 and Table 1 systematically compare the mechanical behavior of CG and FG samples under quasi-static tensile and HCF conditions. As shown in Fig 2a, the FG sample consistently exhibits superior stress response throughout the tensile process, with UTS of 283.7 MPa (an increase of approximately 22%) compared to the CG sample's 231.7 MPa. The YS rises from 134.2 MPa to 164.5 MPa, representing 22.6% improvement. In addition, the EL values are 12.1% for the FG sample and 11.4% for the CG sample, indicating that grain refinement enhances strength without compromising ductility. The strengthening effect induced by grain refinement was further evaluated using the Hall–Petch relationship (σ₀ + kd^-1/2^) [27], where σ_0_ represents the lattice friction stress, k is a constant, and d is the average grain size. Using σ₀ = 124 MPa and k = 130 MPa·μm^1/2^ [30], the predicted yield strength increases from ~140.5 MPa for the CG sample to ~170 MPa for the FG sample, corresponding to an increase of ~21%, which agrees well with the experimental result (~22.6%). This confirms that the observed strengthening is quantitatively consistent with grain size refinement.

**Table 1:** The tensile and fatigue properties of the CG and FG samples.

Sample	UTS / MPa	YS / MPa	EL / %	σ_-1_ / MPa	σ_-1_ / UTS	$\overset{\prime }{\underset{f}{\sigma }}\;/\;$MPa	*b*
CG	231.7 ± 3	134.2 ± 4	11.4 ± 0.5	80	0.34	241.6	−0.072
FG	283.7 ± 5	164.5 ± 3	12.1 ± 0.6	110	0.39	333.8	−0.065

Fig 2b presents the S–N curves illustrating the fatigue behavior of the two samples under HCF loading. Across all cycle regimes, the FG sample consistently exhibits higher stress amplitudes than the CG sample, with the difference becoming particularly pronounced in the 10^5^ to 10^6^ cycle range, demonstrating the superior fatigue endurance of the FG sample. A fitting analysis based on the Basquin equation, $\sigma_{-\text{1}}=\;\overset{\prime }{\underset{f}{\sigma }}{\left(\text{2}N\right)}^{b}$ [13,35], was performed on the fatigue data. The fitted parameters in Table 1 further confirm this trend, the fatigue strength σ ₋ ₁ of the FG sample reaches 110 MPa, a 37.5% increase over the CG sample’s 80 MPa. The fatigue strength coefficient $\overset{\prime }{\underset{f}{\sigma }}$ increases from 241.6 MPa to 333.8 MPa, reflecting greater resistance to fatigue damage in the high-stress amplitude regime [17]. The Basquin exponent *b* for FG is –0.065, higher than –0.072 for CG sample. According to Pan et al. [17] reports, higher values of $\overset{\prime }{\underset{f}{\sigma }}$ and *b* suggest enhanced resistance to cyclic plastic strain localization and surface roughening, both of which contribute to improving σ ₋ ₁. These findings reinforce the beneficial role of grain refinement in elevating fatigue resistance. Moreover, changes in the Basquin parameters not only reflect the differences in fatigue limit but also indirectly reveal the underlying mechanisms of microcrack initiation. The higher $\overset{\prime }{\underset{f}{\sigma }}$ and *b* values for FG sample suggest that the sample is more effective at suppressing early fatigue damage accumulation and surface crack nucleation under high-stress conditions [36], thereby extending fatigue life.

In summary, grain refinement significantly enhances both the tensile strength and fatigue resistance of AZ81 Mg alloy, effectively broadening its application window in structural contexts. This is particularly advantageous in engineering scenarios that demand a synergy of high strength and long service life, such as lightweight components in sports equipment.

### 3.3. Fatigue cracking behavior

**Fig 3:**
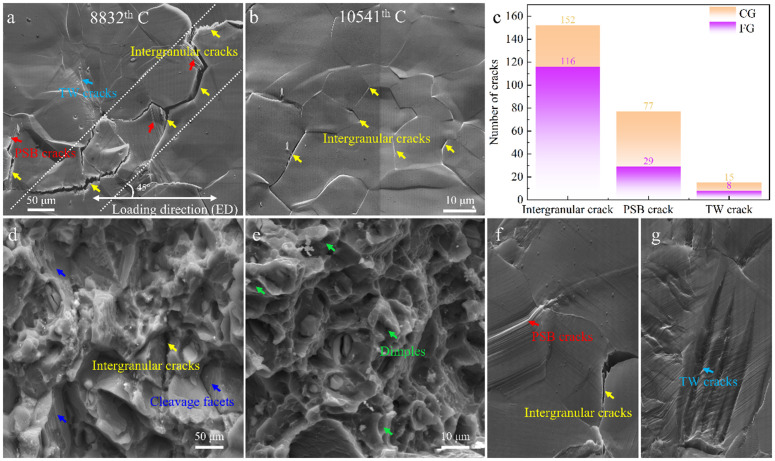
Fatigue damage morphology of the CG and FG samples at Δσ/2 = 130 MPa: (a, d) CG sample and (b, e) FG sample, showing surface crack morphology and corresponding fracture features, respectively, (c) statistics of fatigue cracking incidents, (f, g) representative examples of crack type identification.

Fig 3 shows the fatigue surface morphologies of CG (Fig 3a) and FG (Fig 3b) samples after undergoing 8,832 and 10,541 cycles, respectively, under a stress amplitude of 130 MPa. In the images, yellow arrows indicate intergranular cracks, red arrows denote persistent slip band (PSB) cracks, and cyan arrows highlight twin (TW) cracks. Fig 3c summarizes the number and proportion of each crack type for both samples, revealing distinct differences in their fatigue damage evolution mechanisms. To ensure reliability, the statistical analysis covers an area containing at least 800 grains. In the CG sample, a total of 244 cracks were identified, with intergranular cracks accounting for 62.3% (152 cracks), PSB cracks 31.6% (77 cracks), and TW cracks 6.1% (15 cracks). In contrast, the FG sample exhibited 153 total cracks, including 116 intergranular cracks (75.8%), 29 PSB cracks (18.9%), and only 8 TW cracks (5.2%). These results indicate that while intergranular cracking dominates in both microcracks, the CG sample exhibits significantly more PSB- and twin-induced cracks, reflecting higher levels of localized plastic deformation and deformation heterogeneity. Fracture surface morphologies of the CG and FG samples were further analyzed using SEM, as shown in Fig 3d and e. The CG sample exhibits typical quasi-cleavage features, characterized by the coexistence of tear ridges and cleavage facets, along with a small number of dimples. In contrast, the FG sample shows tear ridges together with a large number of dimples, indicating a mixed fracture mode consisting of quasi-cleavage and ductile fracture.

From the morphological perspective, the CG sample features crack bands inclined at approximately 45° (Fig 3a), which is consistent with the typical growth direction of fatigue cracks along the maximum shear stress plane [13]. PSB and TW cracks frequently initiate within grains or near grain boundaries where slip or twinning activity is pronounced, indicating that coarse-grained microstructures are more prone to activate multiple deformation mechanisms, thereby promoting multi-site crack initiation. In contrast, grain refinement in the FG sample effectively suppresses the formation of PSBs and twins, resulting in predominantly intergranular cracking with significantly reduced crack density. Cracking types on the fatigue fracture surfaces were identified based on their characteristic morphological features. PSB-induced cracks were characterized by persistent slip markings and localized slip bands, while twin-related cracks were associated with twin boundaries and exhibited lamellar features. Intergranular cracks were identified by their propagation along grain boundaries, typically showing relatively smooth or faceted morphologies. Representative examples of these crack types are provided in Fig 3f and g.

### 3.4. Local strain distribution

**Fig 4:**
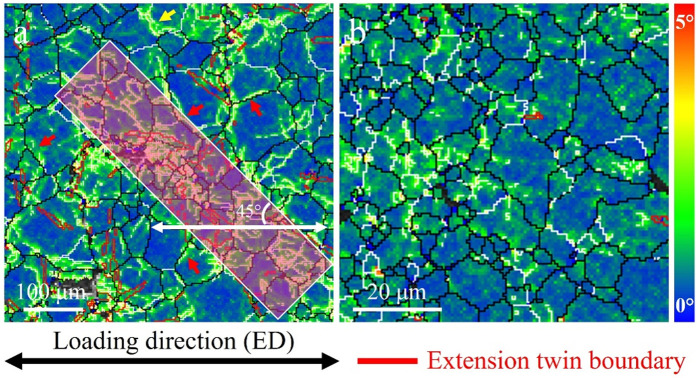
Kernel average misorientation (KAM) maps: (a) CG, and (b) FG samples.

Fig 4 presents the Kernel Average Misorientation (KAM) maps of CG (Fig 4a) and FG (Fig 4b) samples after 8,832 and 10,541 cycles, respectively, under a stress amplitude of 130 MPa, to reflect the distribution of localized strain during fatigue deformation. KAM values quantify local lattice orientation deviations and are widely used to assess dislocation density and the extent of plastic deformation [37,38]. In the maps, color gradients from blue to red indicate increasing local strain, while red arrows highlight pronounced regions of strain localization, typically located near grain boundaries or twin bands.

In the CG sample (Fig 4a), the KAM map reveals significant strain heterogeneity, with large bands of high KAM values oriented approximately 45° to the loading direction. These regions indicate intense dislocation accumulation and active slip, characteristic of plastic zones. High-strain areas are often associated with PSB and twinning activity and serve as potential fatigue crack initiation sites [39–42]. Notably, many of these strain-concentrated zones are found near grain boundaries or across twin boundaries, suggesting that such interfaces act as strong obstacles to dislocation motion, promoting localized stress concentration and crack initiation. In contrast, the FG sample (Fig 4b) displays a much more uniform strain distribution with no apparent high-strain bands, indicating that its grain boundaries more effectively constrain dislocation motion and enhance slip compatibility. This suppresses the activation of multiple crack initiation sites and improves fatigue resistance. The KAM results thus highlight the distinct differences in fatigue deformation mechanisms between CG and FG samples. While coarse grains promote early crack initiation due to strain localization, fine grains enable a more homogeneous deformation response, significantly enhancing resistance to fatigue damage.

### 3.5. Dislocation configuration

**Fig 5:**
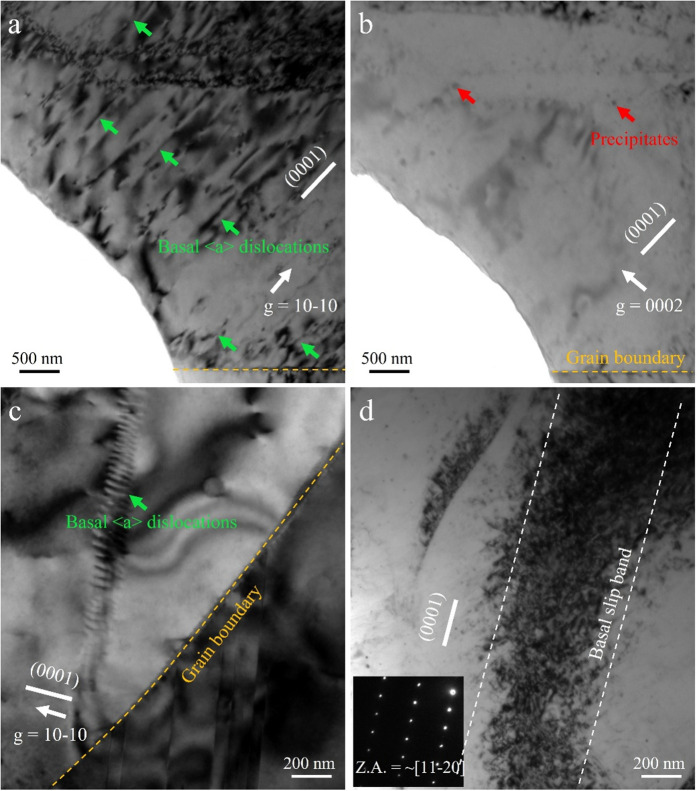
Typical dislocation configuration after fatigue at Δσ/2 = 130MPa in the CG sample: (a), and (c) TEM images viewed with *g* = 10$\overset{―}{1}$0, (b) TEM images viewed with *g* = 0002, (d) TEM image of grain taken along ~[11$\overset{―}{2}$0] axis.

Fig 5 shows the microstructural features of the CG sample after cyclic loading under a stress amplitude of 130 MPa during HCF, revealing the dominant fatigue deformation mechanisms. Fig 5a and b are bright-field images captured under two-beam diffraction conditions. Fig 5a taken with diffraction vector ***g*** = 10−10, clearly shows numerous slip lines and dislocation structures aligned along the (0001) plane, indicating that basal <a> slip (indicated by green arrows in Fig 5a) is the primary deformation mode in the CG sample under fatigue loading. Additionally, dense dislocation pile-ups are observed near grain boundaries (indicated by yellow dashed lines), suggesting that these boundaries strongly impede dislocation motion, resulting in local stress concentration that can promote crack initiation. Fig 5c further confirms dislocation behavior, showing well-defined slip bands and high-density basal dislocations near grain boundaries. These features reflect the obstruction of slip transfer at the boundaries, leading to dislocation accumulation and localized hardening [41]. However, no significant ⟨c + a⟩ dislocations are observed in the Fig 5b (taken with ***g*** = 0002), indicating that <c + a> slip systems were not activated under the current fatigue conditions and that basal <a> slip remained dominant.

Moreover, as indicated by the red arrows in Fig 5b, a small number of dispersed nano-sized precipitates are present within the sample, likely formed via dynamic precipitation during fatigue [8]. Although these precipitates can act as pinning sites for dislocations, their limited quantity suggests a weak contribution to cyclic strengthening. In addition, Fig 5d reveals a well-defined basal slip band enclosed by a white dashed line (captured near the zone axis ~ [11–20]). The slip band contains a high dislocation density and a distinct banded morphology, confirming the localization of slip activity under HCF loading. These slip bands are often closely associated with crack initiation sites and correspond well to the PSB crack locations observed in Fig 3.

**Fig 6:**
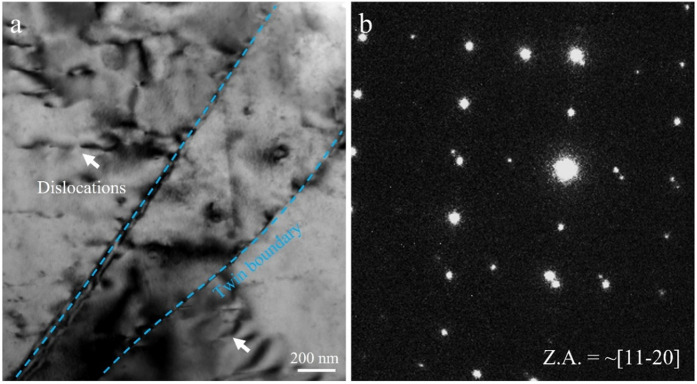
Typical extension twin structure in the CG sample: (a) bright field-TEM image along the ~ [11–20]_α_ zone axis, (b) the corresponding SAED pattern of the (a).

Fig 6 further reveals the microstructural deformation behavior of the CG sample under HCF, with a focus on the interaction mechanisms between twinning and dislocations during fatigue. Fig 6a displays a typical extension twin structure, with the twin boundary clearly marked by cyan dashed lines. On both sides of the twin boundary, numerous dislocations pile-up can be observed (indicated by white arrows). This indicates that twin boundaries act not only as a deformation coordination mechanism during fatigue but also serve as effective barriers to dislocation motion, leading to localized stress concentration [16]. Such dislocation pile-up at interfaces is commonly associated with fatigue crack initiation. Additionally, evidence of dislocations within the twin interior is visible, suggesting that the twinned region also contributes to plastic deformation during cyclic loading. Combined with the TW cracks observed in Fig 3, it is evident that twin boundaries in CG samples can serve as potential crack initiation sites.

The fatigue deformation in the CG sample is primarily governed by basal <a> slip, accompanied by the formation of twins and dislocation pile-ups at twin boundaries. The accumulation of dislocations, formation of slip bands, and the role of twin boundaries as barriers and stress concentrators collectively explain the high density of fatigue crack initiation sites and the inhomogeneous strain distribution observed in the CG sample. The deformation characteristics observed in the CG sample are consistent with previous studies on coarse-grained Mg alloys under high-cycle fatigue conditions [43]. It has been widely reported that basal <a> slip dominates plastic deformation in coarse-grained Mg alloys due to its relatively low CRSS, leading to pronounced strain localization and the formation of persistent slip bands [23,44]. Similar observations have been reported in earlier studies, where intense basal slip activity and limited activation of non-basal systems were identified as key factors contributing to premature crack initiation and reduced fatigue resistance [45]. Therefore, the present results further confirm that the dominance of basal slip and the associated strain incompatibility play a critical role in governing fatigue damage in CG Mg alloys.

**Fig 7:**
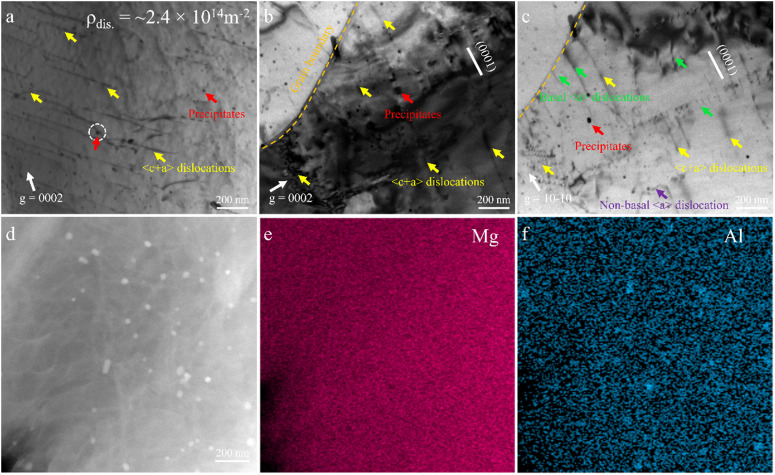
Typical dislocation configuration and precipitates after fatigue at Δσ/2 = 130MPa in the FG sample: (a), and (b) TEM images viewed with *g* = 0002, (c) TEM images viewed with *g* = 10$\overset{―}{1}$0, (d) STEM image, (e) and (f) EDS maps.

Fig 7 systematically presents the deformation microstructure features of the FG sample under a stress amplitude of 130 MPa after fatigue. Fig 7a is a bright-field TEM image obtained under ***g*** = 0002 diffraction conditions. Several parallel dark and bright line features are identified as <c + a> dislocations (yellow arrows), with long slip traces and dense distribution, indicating that non-basal slip systems are significantly activated in the FG sample. The dislocation density was quantitatively estimated using the line-intercept method according to ρ = 2Nₗ / t_foil_, where Nₗ is the number of dislocation intersections per unit length and t_foil_ is the TEM foil thickness determined by CBED [46]. In the present study, Nₗ is approximately 1.2 × 10⁷ m ^−^ ¹ (~12 intersections per μm), and t_foil_ is about 100 nm, dislocation density of ~2.4 × 10¹⁴ m ^−^ ². This relatively high dislocation density indicates intense dislocation activity during cyclic deformation. More importantly, the observed dislocation configurations are dominated by <c + a> dislocations and multiple slip systems, rather than single basal slip. Compared with typical coarse-grained Mg alloys, where deformation is often dominated by basal <a> slip with strong strain localization, the higher density of non-basal dislocations in the FG sample suggests enhanced activation of additional slip systems. This contributes to improved strain compatibility and reduces local stress concentration [47,48].

In addition, numerous nano-sized precipitates are indicated by red arrows (Fig 7a) throughout the image, suggesting pronounced dynamic precipitation during the fatigue process. Dislocations are observed to bend around these precipitates, forming loops consistent with the Orowan bypass mechanism, in which dislocations bow out and circumvent non-shearable hard particles. The presence of Orowan loops confirms that the precipitates act as strong obstacles to dislocation motion, increasing slip resistance and thereby improving fatigue strength [49]. Compared to the CG sample, the FG microstructure contains a greater number of nano-sized precipitates. The formation of these particles is primarily attributed to the stress fields around dislocations lowering the nucleation barrier for precipitation. Fig 7b further confirms the local deformation mechanisms. Under ***g*** = 0002 imaging conditions, abundant <c + a> dislocations are clearly visible, many of which originate from or terminate at grain boundaries (marked by brown dashed lines), indicating that grain boundaries act not only as barriers but also as active sites for dislocation transmission. Fig 7c reveals the concurrent activation of multiple slip systems in the FG sample (taken under ***g*** = 10−10 conditions). In addition to <c + a> dislocations (indicated by yellow arrows in Fig 7a-c), basal <a> dislocations (indicated by green arrows in Fig 7c) and non-basal <a> dislocations (indicated by purple arrows in Fig 7c) are also observed, demonstrating the coordinated activation of various slip systems in the FG sample. Furthermore, the presence of a large number of uniformly dispersed nanoscale precipitates effectively suppresses dislocation motion, enhancing the cyclic stability and crack initiation resistance of the FG sample. To further identify the nano-sized precipitates, STEM imaging combined with EDS analysis was performed (Fig 7d-f). The results show that the precipitates are enriched in Mg and Al, suggesting that they correspond to Mg–Al phases, most likely Mg_17_Al_12_, which is commonly observed in Mg–Al alloys [34].

From a mechanistic perspective, grain refinement and precipitation strengthening play distinct roles. Grain refinement primarily alters the deformation behavior by promoting non-basal slip activation and improving strain compatibility. In contrast, the nano-sized precipitates act as obstacles to dislocation motion, thereby enhancing cyclic stability. The combined effect of these two factors contributes to the improved fatigue performance of the FG sample.

Grain refinement significantly increases grain boundary density, and these boundaries act as effective barriers that generate higher local stresses, thereby promoting the activation of non-basal slip systems, such as <c + a> dislocations, to maintain plastic deformation continuity. Due to their high critical resolved shear stress (CRSS), < c + a> dislocations are typically difficult to activate in the CG sample [50], however, in the FG sample, dislocation pile-up and stress concentration at boundaries facilitate their activation [8]. The concurrent activation of multiple slip systems enhances strain compatibility and reduces localized plastic strain concentration, effectively delaying crack initiation [51,52]. Additionally, the increased grain boundary density in the FG sample disrupts the continuity of intergranular crack propagation paths and particularly impedes the development of intergranular cracks [53]. Furthermore, the high stress gradients near grain boundaries promote dynamic precipitation, and the resulting nano-sized precipitates can pin dislocations and stabilize slip band structures, further enhancing microstructural stability under cyclic loading [54,55]. These observations are consistent with previous studies on fine-grained Mg alloys [56], where grain refinement has been widely reported to promote non-basal slip activity, improve strain compatibility, and suppress strain localization [27]. Therefore, the combined effects of enhanced <c + a> dislocation activation, improved strain coordination, inhibited crack initiation and propagation, and precipitation strengthening collectively account for the superior fatigue performance of the FG sample.

Overall, the present TEM observations are in good agreement with the general understanding of deformation mechanisms in Mg alloys under cyclic loading. Coarse-grained structures tend to deform via basal slip-dominated mechanisms, leading to strain localization and early crack initiation, whereas grain refinement promotes the activation of multiple slip systems, improves strain compatibility, and enhances resistance to fatigue damage. The additional contribution of dynamic precipitation further stabilizes dislocation structures and impedes crack development. These combined effects highlight the critical role of microstructural refinement in tailoring fatigue performance.

## 4. Conclusions

(1) The FG sample exhibits superior quasi-static strength and high-cycle fatigue performance compared to the CG sample, with the yield strength increasing from 134.2 MPa to 164.5 MPa (~22.6%), and the fatigue strength at 10⁶ cycles from 80 MPa to 110 MPa (~37.5%).
(2) Grain refinement suppresses strain localization and reduces PSB- and twin-induced crack initiation by promoting the activation of non-basal <c + a> slip systems, thereby enhancing strain compatibility and delaying crack initiation.
(3) The synergistic effects of increased grain boundary density and dynamic precipitation (e.g., Orowan strengthening) effectively hinder dislocation motion and crack propagation, providing the underlying mechanism for the enhanced fatigue resistance of the FG sample.

## Supporting information

S1 Fig Distribution of Schmid factor for basal slip mode for CG and FG sample. (DOCX)
