# Supplementary figures and images for: Mechanistic origin of high-cycle fatigue enhancement by grain refinement in AZ81 magnesium alloy for sports equipment
Source: PLoS One. 2026 Jun 4;21(6):e0350435. doi: 10.1371/journal.pone.0350435 (PMC13235908; doi:10.1371/journal.pone.0350435)

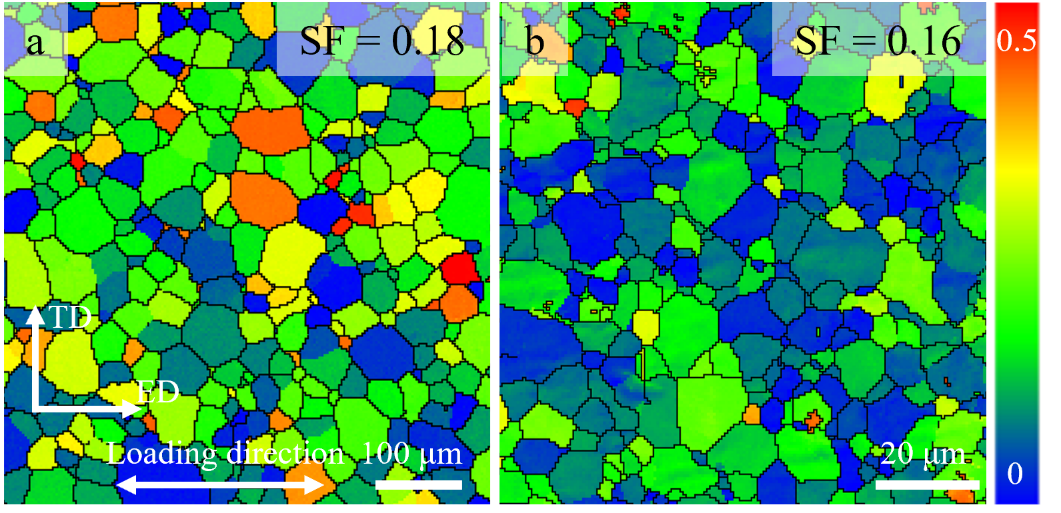


**Fig.S1.** Distribution of Schmid factor for basal slip mode for CG and FG sample.

Supplement: S1 Fig — (DOCX) [file pone.0350435.s001.docx]
